# A Systematic Study on Long-acting Nanobubbles: Current Advancement and Prospects on Theranostic Properties

**DOI:** 10.34172/apb.2024.042

**Published:** 2024-03-17

**Authors:** Gokulnath Jayasankar, Jebastin Koilpillai, Damodharan Narayanasamy

**Affiliations:** Department of Pharmaceutics, SRM College of Pharmacy, SRM Institution of Science and Technology, Kattankulathur, Chengalpattu, India.

**Keywords:** Nanobubble, Long-acting, Theranostic, Tumor, Contrast agent

## Abstract

Delivery of diagnostic drugs via nanobubbles (NBs) has shown to be an emerging field of study. Due to their small size, NBs may more easily travel through constricted blood vessels and precisely target certain bodily parts. NB is considered the major treatment for cancer treatment and other diseases which are difficult to diagnose. The field of NBs is dynamic and continues to grow as researchers discover new properties and seek practical applications in various fields. The predominant usage of NBs in novel drug delivery is to enhance the bioavailability, and controlled drug release along with imaging properties NBs are important because they may change interfacial characteristics including surface force, lubrication, and absorption. The quick diffusion of gas into the water was caused by a hypothetical film that was stimulated and punctured by a strong acting force at the gas/water contact of the bubble. In this article, various prominent aspects of NBs have been discussed, along with the long-acting nature, and the theranostical aspect which elucidates the potential marketed drugs along with clinical trial products. The article also covers quality by design aspects, different production techniques that enable method-specific therapeutic applications, increasing the floating time of the bubble, and refining its properties to enhance the prepared NB’s quality. NB containing both analysis and curing properties makes it special from other nano-carriers. This work includes all the possible methods of preparing NB, its application, all marketed drugs, and products in clinical trials.

## Introduction

 Nanotechnology-based medicines refer to pharmaceuticals that use nanotechnology to treat, detect, or prevent certain diseases. It has shown an increased potential for therapeutic efficacy and lowering the side effects.^1-4^ Research related to nanomedicine prepares the ground for the development of novel Nano-biomaterials and Nanotherapeutics and shows a drastic improvement in diagnostics, contrast agents, and medical devices. The ideology of Nanomedicine originated in the late 1990s.^5-13^

**Figure 1:**
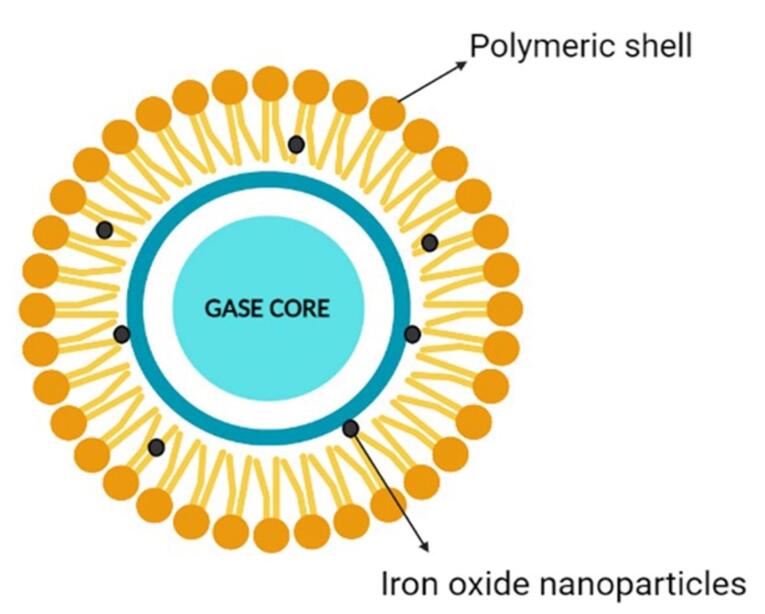
Nanobubble an overview

 Nanobubbles (NBs) are sphere-shaped particles containing an outer covering shell and a vapor-filled core composition that can exhibit dynamic features (Figure 1). They are gas-carrying concave voids in an aqueous solution that range in size from below 1 nm.^14-16^ The majority of these shells are constructed of lipids, proteins, surfactants, and multilayers of polyelectrolytes, while the core foundation can be made from a variety of gases, including perfluorocarbon, carbon dioxide (CO_2_), sulfur hexafluoride, and air.^17^ By laying the path for leakage from blood arteries to the tissues around it, NBs enhance the drug’s transport and adaptation.^18^ NBs provide a lengthy lifetime for experimental examination with Epstein and Plesset’s hypotheses.^19^

 This Nano system was initially designed as a contrast agent, effectively reflecting ultrasound (US) energy, manufacturing asymmetric oscillation in diameters when disclosed to the auricular field. On top of that, it also shows interest in medication delivery, gene therapy and gas delivery.^20,21^ NBs associated with US are frequently employed in medicine delivery. Nanoparticles may serve as medicinal cavitation sites for sonoporation caused by US, generating brief gaps in membranes in order to alter cell susceptibility. To achieve this goal, NBs could be used as US-mediated nano cargo to deliver pharmaceuticals into cells or a medication may be included into the bubble structure.^22^ NBs offer the potential to change the properties of water; they are smaller and more focused than other bubbles and are unable to float because of their low density due to their small diameter.^23^

**Figure 2:**
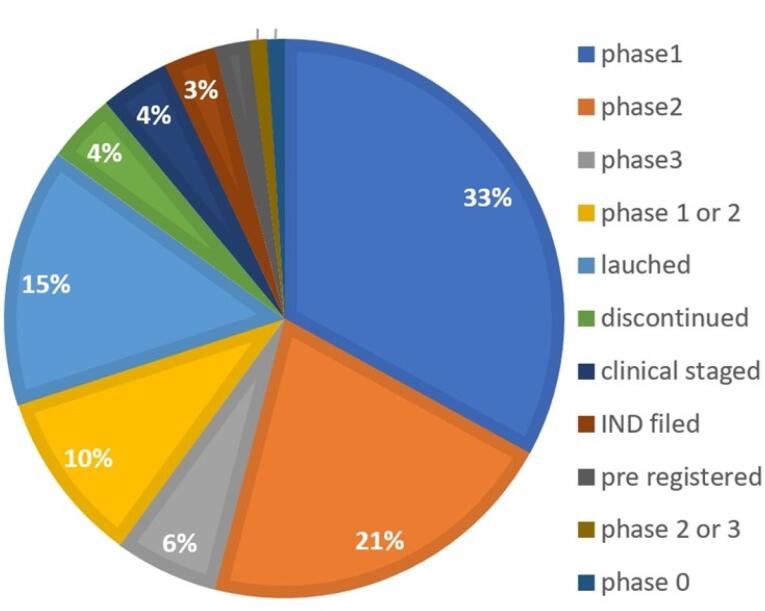
Available nanobubbles in sales or in clinical trials- Development status

**Figure 3:**
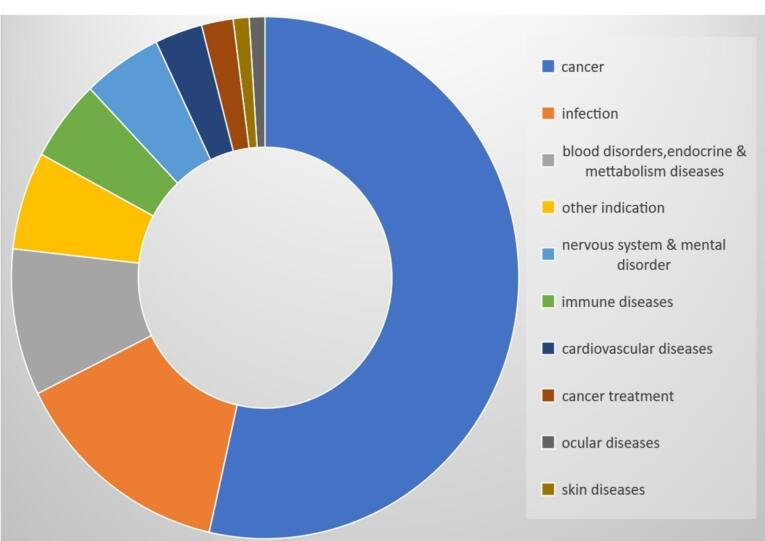
Available nanobubbles in sales or in clinical trials- Indication

**Figure 4:**
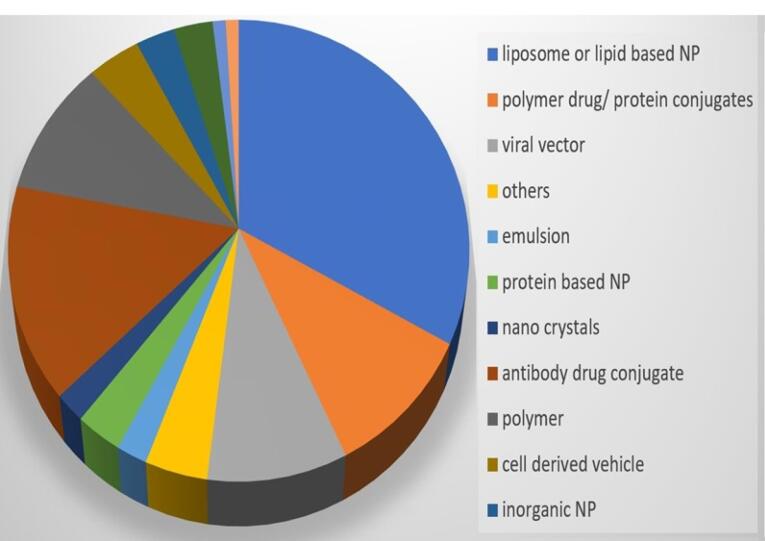
Available nanobubbles in sales or in clinical trials- Formulation

 NBs being Nano-carriers show a variety of functions in many industries and one of the emerging properties among them is the theranostic activity. Theranostic refers to the integration of diagnostic therapeutic functionalities into a single entity, enabling simultaneous monitoring and treatment of diseases. The unique properties of NBs make them suitable for various applications in theranostics. Usually theranostic agent is a system that gives support to treatment.^24-27^ NBs theranostic hold great promise in various fields including cancer therapy, cardiovascular disease, and regenerative medicine. Despite the fact that there has been a lot of advancement in the field, more work needs to be done in order to perfect the formulation of NBs, enhance targeting techniques, and guarantee their safety and effectiveness in clinical settings (Figure 2). NBs have received vast attention for being theranostic and have evolved in various fields, especially in cancer (Figure 3).^28^ The term theranostic was coined by Funkhouser in the year 2002 and was stated as the material that shows the ability to combine therapy & diagnostic imaging.^29^ NBs accessible in the market or studied under clinical trials are enlisted using pie charts (Figure 4). The main aim of this review work is to showcase the aspiring potential and unique nature of NBs in different routes of administration. Various benefits are attained by using NB formulation and different kinds of methods in preparing NBs. NBs offer enhanced drug delivery capabilities, serving as carriers for therapeutic agents and enabling targeted delivery to specific tissues or cells. Their ability to penetrate biological barriers more effectively than conventional drug delivery systems leads to improved drug efficacy and reduced side effects.^30^ Additionally, NBs can enhance the solubility of poorly water-soluble drugs, providing a high surface area for dissolution and thereby improving bioavailability and therapeutic effectiveness. Controlled release mechanisms can be engineered into NBs, allowing for sustained or triggered drug release profiles, which improves drug stability, reduces dosing frequency, and enhances patient compliance.^31^ Moreover, NBs find utility in diagnostic imaging as contrast agents for modalities such as US and magnetic resonance imaging (MRI), enhancing resolution and sensitivity for early disease detection and monitoring. Despite these advantages, NB formulations face challenges such as stability issues, scale-up difficulties, and complex characterization requirements. Maintaining stability, especially in physiological conditions, necessitates specialized stabilizers or encapsulation techniques.^32^ Scaling up production from laboratory-scale to industrial-scale poses challenges in reproducibility, consistency, and cost-effectiveness. Furthermore, characterizing NBs and navigating regulatory hurdles for clinical or commercial use can be complex and expensive. Cost considerations, including the use of specialized equipment and materials, may limit widespread adoption, particularly in resource-limited settings. Therefore, while NB formulations offer promising opportunities, addressing these challenges is essential for realizing their full potential in pharmaceutical applications.

 The study provides a comprehensive overview of NBs and their potential applications in pharmaceuticals. It covers their composition, preparation methods, therapeutic drug loading, and shell types. One notable aspect is the emphasis on the therapeutic potential of NBs in drug delivery and cancer therapy. By encapsulating therapeutic agents within the core or shell of NBs, they can be targeted to specific tissues or cells, enhancing drug efficacy while minimizing side effects. Moreover, the study discusses the importance of shell composition in determining the stability and functionality of NBs. Various materials like lipids, proteins, and polymers are explored for shell construction, highlighting their impact on NB properties. Understanding this role is crucial for optimizing NB performance in biomedical applications. Additionally, the incorporation of Quality by Design (QbD) principles in NB development is noteworthy. By identifying critical quality attributes (CQAs) and critical material attributes (CMAs), researchers can systematically optimize NB formulations and manufacturing processes to meet desired specifications. Furthermore, the study delves into novel preparation techniques for generating NBs, such as electrocatalytic generation and ultrasonic cavitation. These approaches offer alternative methods to traditional ones and may lead to more efficient and scalable NB production processes. Addressing challenges associated with NB characterization, such as their small size and transient nature, the study proposes innovative solutions. By employing specialized analytical methods and scaling up production processes, researchers can ensure reproducibility and quality in NB formulations. Overall, the study contributes to advancing NB technology and its translation into clinical practice by providing insights into formulation, preparation, and characterization challenges, along with innovative solutions to overcome them.

## Composition of NBs

 Various methods are employed for the preparation of core-shell NBs tailored for specific applications in the biomedical field. In tumor imaging, octafluoropropane is encapsulated within 1,2-stearoyl-sn-glycerol-3-phosphatidylcholine (DSPC) using centrifugation. For cancer therapy, oxygen is encapsulated within dioleoyl phosphatidylcholine (DOPC) via emulsification. In cardiac transplants for identifying acute rejection, anti-CD3 antibody is encapsulated within DSPC, DSPE-polyethylene glycol (PEG) 2000, and biotinylated DSPE-PEG2000 using the thin-film hydration method. Similarly, oxygen is encapsulated within lecithin via sonication to enhance the effectiveness of existing cancer medicines and decrease tumor oxygen levels. Chitosan is utilized with oxygen via ultrasonic high-shear mixing for lung cancer treatment. Imaging of atherosclerotic plaque involves a gas core encapsulated within 1,2-dimyristoyls-sn-glycerol-3-phosphocholine (DMPC) using the ultrasonic emulsion method. Targeting molecules to the posterior regions of the vitreous humor is achieved through encapsulation of rhodamine-tagged perfluoropropane gas within 1,2-dipalmitoyl-sn-glycerol-3-phosphocholine (DPPC), 1,2-stearoyl-sn-glycerol-3-phosphoethanolamine-N-[methoxy(PEG)-2000], and 5-carboxy-tetramethylrhodamine N-succinimidyl ester (5-TAMRA) via the emulsion method. For tumor imaging and theranostic purposes, perfluoropropane gas is encapsulated within PLA-PEG-NH2 block copolymers using centrifugation. Microfluidics is utilized for encapsulating perfluorobutane within phospholipids to induce medication release under US stimulation. Mechanical agitation is employed for encapsulating octafluoropropane within phospholipids for US-triggered drug release and gene delivery in cancer treatment. Similarly, liposomes are prepared via sonication for localized gene delivery, while chitosan is utilized with 2H,3H-decafluoropentane using the double emulsion method for gene delivery in melanoma treatment. Oxygen and mitomycin are encapsulated within hydrogels via sonication for gas delivery. Oxygen encapsulated within dextran via the emulsion solvent method is utilized for hypoxia treatment. Furthermore, octafluoropropane is encapsulated within phospholipids using mechanical agitation and PEG40S & SPAN60 via sonication for US imaging (Table 1).

**Table 1:** NBs based on their preparation & application

**Core**	**Shell**	**Preparation method**	**Application**	**Reference**
Octafluoropropane	1,2-Stearoyl-sn-glycerol-3-phosphatidylcholine (DSPC)	Centrifugation	Tumor Imaging	^33^
Oxygen	Dioleoyl phosphatidylcholine (DOPC)	Emulsification	Cancer Therapy	^34^
anti-CD3 antibody	DSPC, DSPE-polyethylene glycol (PEG)2000 and biotinylated DSPE-PEG2000	Thin-film hydration method	In cardiac transplants, identify acute rejection	^35^
Oxygen	Lecithin	Sonication	To improve the effectiveness of currently available cancer medicines, decrease tumour oxygen.	^36^
Oxygen	Chitosan	Ultrasonic High Sher Mixing	Lung Cancer	^37^
Gas core	1, 2-Dimyristoyls-sn-glycerol-3- phosphocholine (DMPC)	Ultrasonic emulsion Method	Imaging of atherosclerotic plaque	^38^
Rhodamine-tagged Perfluoro propane gas	1,2-dipalmitoyl-sn-glycerol-3- phosphocholine (DPPC), 1,2-stearoyl-sn-glycerol-3- phosphoethanolamine-N- [methoxy(PEG)- 2000] and 5-carboxy-tetramethylrhodamine N-succinimidyl ester (5-TAMRA)	Emulsion method	Targeting molecules to posterior regions of the vitreous humor	^39^
Perfluoro propane gas	PLA-PEG-NH2 block copolymers	Centrifugation	Tumour Imaging and Theranostic	^28^
Perfluorobutane	Phospholipid	Microfluidics	medication release caused by US	^32^
Octafluoropropane	Phospholipid	Mechanical agitation	US triggered drug release	^40^
Oxygen	Lipids	Sonication	medication release caused by US	^41^
Octafluoropropane	Phospholipid	Mechanical agitation	Gene delivery treating cancer	^42^
Octafluoropropane	Liposome	Sonication	Localized gene delivery	^43^
2H,3H-Decafluoropentane	Chitosan	Double emulsion method	Gene delivery treating melanoma	^44^
Oxygen & mitomycin	Hydrogel	Sonication	Gas delivery	^45^
Oxygen	Dextran	Emulsion solvent	Treatment of hypoxia by oxygen delivery	^46^
Octafluoropropane	Phospholipid	Mechanical agitation	US imaging	^47^
Octafluoropropane	PEG40S & SPAN60	Sonication	US imaging	^48^

**Figure 5:**
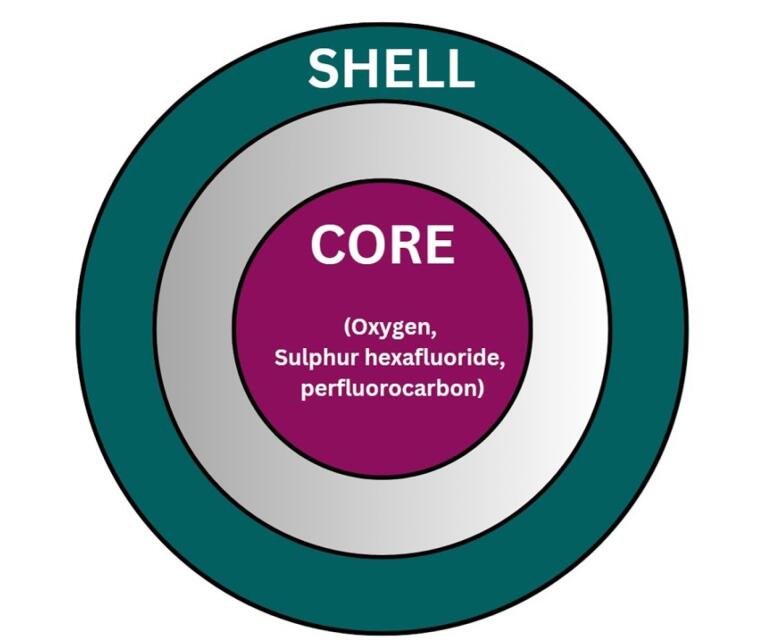
Composition of NB

 Here are the two primary types of NB constituents—the inner core and the outside shell—with their various physicochemical characteristics (Figure 5). The shell predominantly is composed of surfactants, polymers, or proteins, and the core air contains sulfur hexafluoride and perfluorocarbon. Secure, bioabsorbable, recyclable, regulatory compliance-approved substances must be selected for system formulation. Shell composition must be taken into account when formulating bubbles. The fatty acids or polymeric materials are often used in the outer covering of NBs to promote durability towards air loss, dissolution, and foaming growth.^49^ The first factor that can significantly affect the bubble life duration is the composition of the shell, which has an impact on how gases are exchanged between the core and the medium outside the bubble. The stability of NBs is governed by the thickness and elasticity of the shell.^50^ Acoustic parameters that trigger drug release depend on the structure of the NBs. In particular, the response of NBs in the sound province is fiercely affected by the springy nature of the shell.^51^ Phospholipids make up the shell that is the most flexible. The properties of the shell can be significantly influenced by the hydrocarbon tails of these lipids. Surface pressure falls and gas permeation resistance rises while the total amount of carbon molecules increases. However, longer carbon chains result in higher shell density, which produces stronger and fewer echogenic bubbles. For example, saturated diacyl chains are more rigid and often less gas-permeable than short-chain lipids. Because of this, NBs made of fatty acids with greater duration acyl chains last longer.^52^ Grishenkov et al proposed that Polyvinyl alcohol was used to generate polymer-shelled microbubbles which showed improved stability compared to conventional ones replacing the bubble with a phospholipid shell.^53^ Chemical or materialistic cross-linking of the shells offers another approach to fabricating stable NB systems in the presence of US.^54^

###  Core

**Figure 6:**
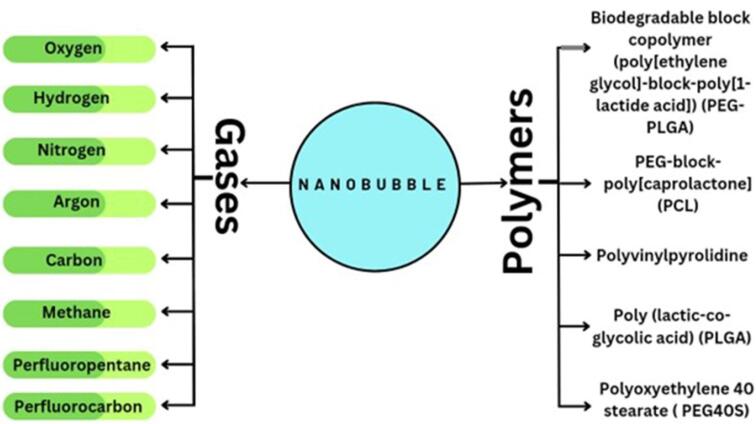
Nanobubble Formation Using Gases and Polymers

 The majority of the particle’s volume is taken up by the core, which is an independent sparsely dense chamber, usually made with medicinal gas (Figure 6). The aqueous solution and surrounding gas densities cause the NBs to vibrate when sound waves are introduced. When the sound pressure is low, the NBs vibrate frequently, causing a steady cavitation phenomenon that accelerates the diffusion of the base gas out of the bubble. For the goal of creating NBs, the gas in the core should be controlled because it is a crucial parameter. The duration and stability of the NBs are increased by combining oxygen with perfluorocarbons and sulfur hexafluoride. Gases such as nitrogen monoxide were also used in the core instead of oxygen. By changing the gas in the core, different uses of NBs can be exploited. For example, oxygen in the core can be used to reverse hypoxemia in the blood and improve blood oxygen levels.^55^

 NBs can be imbued intravenously. Contrast agents require persistent NBs to provide contrast over long periods. Low- to medium-intensity US is used to create bubbles that exist within a firm phenomenon of the cavitation stage for imaging purposes. Numerous researchers have investigated how NBs release oxygen in their pure form. Without the use of US, oxygen NBs instantly collapse to liberate the core gases, particularly for lipid shells.^56^ Using a high molecular weight hydrophobic gas with poor solubility in the NBs allows for improved efficiency.^57^

###  Therapeutic drug loading

 NBs often use three feasible techniques to pile therapeutic doses.^58^ First, a medication is enclosed in the core that lies inside the NBs, perfluorocarbons directly dissolve lipid-based drugs. According to research, adding a co-solvent or oil to its core may assist lipophilic medicines in dissolving.^59^ NBs can transport special gases such as hydrogen (H_2_), nitrogen (N_2_), and oxygen. (O_2_) or nitric oxide (NO) at its core and may be beneficial in conventional medicines solicitation by affecting the corporal and medical etiological processes.^60^ Via an electrostatic connection, drugs can be put within or under the shell.^61^ Repellent drugs can be loaded right into the lipid envelope. The thin monolayer shell, however, prevents a high enough drug incorporation performance. Given that this is an anionic/cationic body, paid components like nucleic acids and doxorubicin can be capacitively attached with ease; nevertheless, this is constrained by uncontrolled drug release.^62^ To prevail over this restriction, multilayer systems seemed to be explored with the chemical bonding of bubbles to the shell surface. For example, a multilayer system that coats the foam with deoxyribonucleic acid (DNA) and polymer has been acclimated sequentially to cohere contrarily charged polyelectrolytes to the facet of the dish.^63^ The genetic material was shielded from enzymatic digestion using a polymer coating. A special multi-layer technology significantly increases the amount of active ingredients. A third approach is to encapsulate the drug in a nanometric material and bind it to the blister shell.

 Additionally, the flexible nature of NBs has been investigated for the simultaneous administration of two or more diverse medications.^64^ Cavalli et al came up with a type of dextran NBs in which affirmatively charged cisplatin is packed into every bladder envelope and soluble doxorubicin is retained inside the vapor gist. Upon irradiation with the US, vesicles loaded with two different drugs were found to be significantly more therapeutically effective against tumor cells.^17^

###  Shell 

 Unbound gas bubbles that aren’t protected by a shell disintegrate swiftly.^65,66^ The outer covering creates a barrier around the gas in order to safeguard its integrity and safety. It guards against internal scavengers and slows the rate at which the core gas diffuses into the adjacent medium.^14,67,68^ If the shell is soft, it will crack easily, but if it is hard, it will be difficult to crack, it vibrates in an ultrasonic field. The shell’s structure affects medication and genetic material loading significantly. As a result, it’s critical to select appropriate shell materials for NBs with a range of thicknesses, stiffness, charges, and functional categories.^69^

###  Lipid-shell

 Since they are sustainable and biodegradable, lipid-based nanoparticles and microparticles are favoured for medicinal purposes. Under sonic pressure, the lipids create pliable shells that are about 3 nm thick. These lipid shells permit the diffusion of gas through them. Since phospholipids are amphipathic and have hydrophilic heads and hydrophobic tails, they are frequently employed for lipid-coated NBs.^69^

###  Protein shell

 Stability, biocompatibility, biodegradable amphiphilicity, and prolonged half-life are traits of protein-coated NBs. The process of creating protein-coated NBs involves emulsifying the protein solution by heating it to the point of denaturation. On the gas of interest, denatured proteins create a fragile monolayer covering. It has been proven that thiol-rich protein molecules like albumin from humans are ideal for binding to create NB shells.^70,71^

###  Polymer shell

 Polymer vesicles have a better drug-loading potential for both hydrophobic and hydrophilic medications due to their thicker—up to 150–200 nm—than lipid and protein walls. When ultrasonic fields are used, polymer envelope bladders tend to be more resistant to stress and expansion. They exhibit small vibrations at low sound pressures, but rupture or damage at higher pressures, allowing gas or agents to diffuse. For quantum imaging, polymer-shell NBs are also chosen due to their outstanding durability and potent US stimulation.^72^

###  Reagents frequently used to create NBs

 Numerous research studies have examined the effects of various ingredient types and the physicochemical characteristics of NB. These substances can be salt and electrolyte solutions, ionic or non-ionized surfactants, or combinations of these. In buoyancy different Surfactants, especially blowing agents, are used to generate and stabilize foam.^73,74^ Pressure, pH, and temperature are additional factors that determine the foam’s characteristics. Reagents’ ability is principally responsible for their stabilizing function. It lessens the air/water interface’s pressure on the surface and surface power.^75^ Surfactants have a great influence on the properties of foam in aqueous solutions. The actual effect of surfactants on NBs generation has been studied in several publications, as their application in flotation (Table 2). In general, we recommend using a good frother by which bubble size can be reduced by 13-35%.^76^

**Table 2:** Properties of excipients used in Nanobubble

**Surfactant**	**Molecular weight**	**Hlb value**	**Reference**
Methyl isobutyl carbinol (MIBC)	102.17	6.00	^75,77–81^
Pine oil (PO)	154.25	5.40	^80^
Dipropylene glycol (DPG)	134.17	9.30	^75^
Dodecylamine (DDA)	185.35	10.70	^82^
Flotigam	195.00	N/A	^83,84^
EDA 3B*			
Dodecylamine hydrocholride (DAH)	221.81	N/A	^85^
PEB70*	-250	N/A	^86^
Dodecyl trimethyl ammonium chloride (DTAC)	263.89	N/A	^87^
Sodium dodecyl sulfate (SDS)	288.37	40	^84,88^
FLO-YS-20*	> 300	N/A	^86^
F507	425.00	8.63	^77^
Polysorbate 80 (tween 80)	1310.00	15.00	^88^

## Stability of NBs

 The astounding lifetime of huge NBs is its most distinctive characteristic. Microbubbles last for just a few moments, but NBs have been seen to last for a longer period of time.^89,90^ Bubbles are known to vibrate when exposed to US. Also, the bladder vibrates differently depending on the applied sound pressure. The mechanical index of ultrasonic devices can be changed to change the sound pressure. Multiply the central frequency of the US by the square root of the US anomalous peak pressure to achieve this. Compared to the normal oscillations of the tissue nearby, this random oscillation represents a distinct signal. When the mechanical index surpasses a critical point, the bubble is destroyed and emits a signal different from the neighbouring tissue in the form of non-linear oscillations for a short time.^91,92^

 In addition, steady cavitation is caused by the nonlinear vibration of bubbles, and inertial cavitation is caused by the destruction of bubbles. Cavitation is accompanied by physical changes in the surrounding cellular structure. In addition, the vapor in each bubble escapes, generating intense shock waves, shear forces, and microjets. These changes include deformation and disruption of vesicles and can induce exchange in lipid plasma membranes even in the absence of inertial cavitation.^93,94^ CO_2_ bubbles have a much shorter lifespan (only 1-2 hours) compared to bubbles due to the much lower pressure in the atmosphere and the ease with which they dissolve in water.^95^ The presence of these surface NBs may be of great technical importance as they have been shown to induce liquid slippage on hydrophobic surfaces, thereby reducing liquid dissipation in small device flows significantly. Observation of long-lived surface Nano bubbles completely contradicts the classical theory of bubble stability.^19,96-98^

###  Cause of instability

 Generally speaking, there are three basic reasons for bubble instability: Thermodynamic instability, devastation from the effects of buoyancy, and coalescence.

 The primary explanation is the surface tension at the gas-liquid interaction, which has an expense in energy. This results in a difference in pressure for spherical bubbles. Laplace pressure is the stress that exists between the inner vapor phase and the outer fluid phase.


$$\Delta P=-\frac{2\sigma }{r}$$

Where, ∆P – Laplace pressure, σ- interfacial tension, r- radius of bubble

 Due to the inverse relationship between bubble radius and surface tension, the predicted pressure on nanoscale bubbles can exceed 3 MPa. The existence of Laplace pressure also leads to a phenomenon known as the “Laplace bubble catastrophe”. In this phenomenon, small fluctuations in bubble size cause bubbles to grow or shrink rapidly.^57,99^ For example, a small increase in bubble size will decrease the Laplace pressure. As described in Henry’s law, this reduces the concentration of gas near the bubble, causing gas to diffuse from the solution into the bubble, creating a positive feedback loop. The same applies to bubbles, which initially decrease in size. This is shown in the Epstein Preset equation.

###  Dynamic equilibrium

 Early attempts to explain the existence of surface Nano bubbles focused on gas transport near the bubbles. A probably trivial explanation for stability is that the bubbles are simply impermeable to gases. An external power supply is required to maintain recirculation flow, but of course, Seddon even suggested that there should be strong (1 m/s) directional currents near the surface of NBs.^100^ According to the principle of dynamic equilibrium, a NB can resist disintegration if a portion of its surface is covered in a hydrophobic substance. A hydrophobic substance repels water, creating a thinner layer on its outer layer. The total amount of the zeta potential (ZP) determines how stable a NB is in gas. The opportunities at the plane of slip interface of the electrical double layer of NBs are referred to as ZP. Higher ZP enriches the firmness of NBs in suspension due to inter-bubble revulsion. Stability and coagulation decrease due to lower ZP. Aggregate NB suspensions’ ZP depends on the electrolyte, surfactant in liquid form, acidity, and gas type. At pH 4–12 and ZP ranges of –4.3 to –62 mV, NBs trapped in this depletion layer are said to be charged down. As an outcome, the water-resistant material’s surface experiences a considerable increase in air pressure above ambient pressure. The skin is made from natural compounds, surfactants, etc., claims the skin theory, completely envelops the exterior of NBs. The entire gas diffusion process from the interior of the NBs to the encircling liquid is blocked.^101,102^

###  Corroboration of nano stability

 Despite the expected unsteadiness, numerous investigations have shown that NBs exist and, in certain situations, that they are slightly more stable than microbubbles. Dynamic light scattering was employed by Dr. Abdalkadar to show that C_3_F_8_ NBs can be kept stable at 4°C for a maximum of 90 hours. However, it is unknown if the percentage of NBs (as contrasted with non-gas particles) remains constant across all experiments due to the nature of dynamic light scattering. It has been proved that the size and concentration of C_4_F_10_ NBs were stable in him for 1 month using nanoparticle tracking analysis. However, similar to dynamic light scattering, We are unable to figure out between non-gas particles and NBs.^103,104^

###  Suggested stability mechanisms

 Due to the fact that NBs have a non-null ZP with a total charge that is independent of the existence of an exterior wetting agent, an electrically charged double layer is present.^105,106^ The electrical double layer on the surface of NBs can induce an electrostatic pressure that opposes the Laplacian pressure. Due to the particles’ magnetic pull on one another, the bilayer also lessens bubble coalescence. A modified Epstein pre-set model that takes into account potential drop was proposed by Tang et al.^107^ Due to the charge conservation supposition of bubbles that contract first, the surface charge density increases and consequently the electrostatic pressure. In an equilibrium state where the electrostatic and Laplacian pressures are equal, the bubble eventually stops contracting. Based on the Debye length and the initial ZP of the bubble, the model estimated a neutral bubble size of approximately 100-300 nm, consistent with findings from dark field microscopy.^108^ In a different investigation, Yin et al discovered that anionic lipid-coated bubbles had a ZP magnitude that diminished from 29.3 mV to 18.36 mV, while the bubble size dropped from 1120 nm to 437 nm.^109^ Increasing concentrations of monovalent and polyvalent salts adversely affect the stability of uncoated NBs, as they reduce the magnitude of the ZP due to electrostatic shielding.^110^ In contrast to predictions of colloidal stability, examination conducted by Michailidi et al. yielded contradictory results indicating an increase in the magnitude of the zeta potential with increasing sodium chloride (NaCl) concentration.^111^ NB endurance may also be influenced by the ‘bulk’ impact of large numbers of NBs and the resulting close space between bubbles. Weijs et al utilized molecular dynamics models, potentially employing the Lennard-Jones approach, to establish that NBs maintain stability when the distance between them remains below a critical threshold.^112^

## Method for preparation of NBs

 The majority of NB production techniques originated from microbubble production methods. The size of the resulting microbubble can be further decreased to make NBs by modifying or enhancing some factors during microbubble formation.

 Thin-film hydration, high-shear emulsification, freeze-drying, and ultrasonic cavitation are currently listed in the literature as the majority of frequently employed techniques for NB preparation (Table 1). Due to the need for micro/NBs in therapeutic uses and the advancement in compound preparation gadgets, novel ways to prepare have evolved to further increase the uniformity and controllability of bubbles, including the microfluidic method and inkjet embossing method.^113,114^ NBs have been measured using various methods for their identification (Table 3).

**Table 3:** NBs characterization

**Parameter**	**Characterization**	**Reference**
Particle size	Scanning electron microscope (SEM), Transmission electron microscope (TEM)	^115^
Bubble size	Dynamic light scattering	^116^
Bubble sized distribution	Tracking analysis	^117^
Physicochemical properties of NBs	Nuclear magnetic resonance (NMR)	^118^
Dissolved oxygen estimation	DO meter	^119^
Electrophoretic mobility of particles	ZetaPALS instrument	^85^
Identifying surface NBs	Atomic force microscopy (AFM)	^120^
Magnetic properties of NBs	Vibrating sample magnetometer (VSM)	^60^
Structure of NBs	Fourier-Transform Infrared Spectroscopy (FTIR)	^117^

###  Sonication method

**Figure 7:**
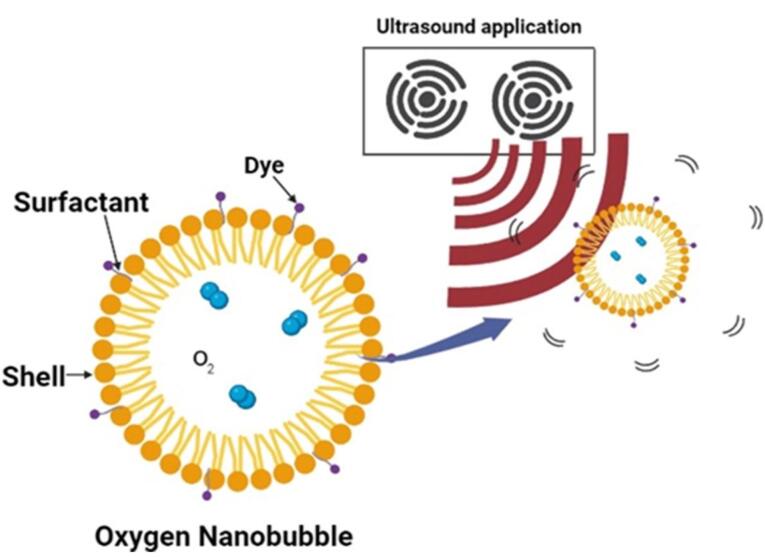
Ultrasound method

 High-intensity ultrasonic waves are used in this technique to move the gas or liquid into a mixture of the appropriate encapsulating material (Figure 7). This technique involves the use of a liquid or gas to produce a suspension of tiny droplets or tiny bubbles. On its surface, proteins and surfactant layers are naturally adsorbed.^18^ Second, due to high temperatures and high pressure, cavitation caused by inertia in suspension changes the outer shell coating material chemically, enhancing its stability by, for example, causing the denaturation of proteins by cross-linking.^121^

###  Emulsification method

 Another well-liked technique for producing NBs, particularly polymer shell bubbles, is high-shear emulsification. High-shear emulsification is another popular method for creating NBs, especially polymer shell bubbles. Water and an additional liquid that is insoluble with the polymer are used as a stabilizing agent, and high-speed spinning is used to emulsify the polymer in a biological solvent-water mixture. Liquid microspheres with a polymer coating form as the biological solvent evaporates on the surface of the particles.^122^

###  Microfluidic method

**Figure 8:**
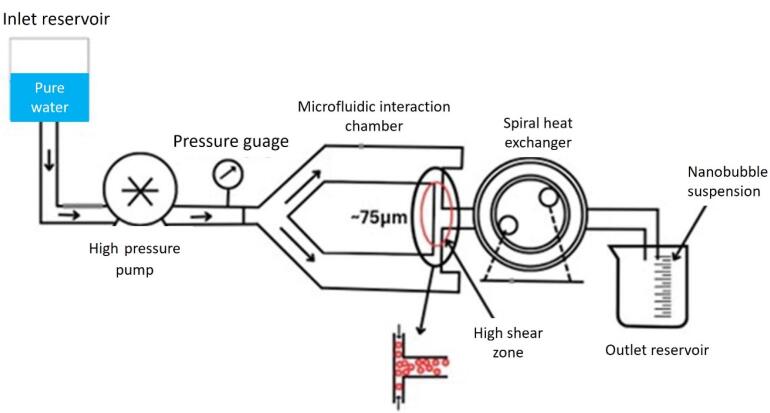
Microfluidic NB generation method

 Microfluidic technology has been used in investigations on the production of NBs (Figure 8). This is because the interaction between gas pressure, fluid circulation rate, and system design in microfluidics enables fine regulation of bubble diameter and generation speed. Gases are continuously injected through lipid, protein, or other shell-like material solutions in microfluidic gadgets at varying flow rates and pressures. Due to this, the gas is retained in the liquid state, grows across the channels, and eventually collapses to create isolated bubbles.^123^ The microfluidic procedure may be completed in a single operation as opposed to previous approaches, and the created bubbles exhibit identical particle sizes and a limited size conveyance. Additionally, by varying preparation factors like velocity and stress, one can alter the amount of medication that is inserted into the bubble envelope, potentially creating multi-layered composite microbubbles. However, microfluidic techniques require relatively more equipment and result in relatively low bubble yields compared to traditional fabrication methods.

###  Ink jet method

 Depending on the application, the polymer solution was pumped through a piezo-driven inkjet nozzle of the necessary size during the creation of microbubbles. In solution, piezoelectric crystals generate pulses and bubbles. Through the opening, the produced liquid is drawn. Through the opening, the produced liquid is drawn. In a similar manner, high-pressure flow via a nozzle was employed to create nanoscale oxygen NBs using water that is pristine and a source of oxygen.^124,125^

###  Laser ablation method

 Aluminium oxide particles in water that have oxidized into nanoparticles can be targeted by an excimer laser of the exact wavelength. At the solid-liquid contact throughout the process, air bubbles are also produced. When a cluster of gold nanoparticles is exposed to a laser pulse of sufficient energy, its temperature rises rapidly, evaporating the surrounding water and forming plasmonic NBs.^126^

###  Agitation method

 This is a promising method for creating NBs on an industrial level. By stirring the solution in a shaker with thousands of vibrations per minute, a lipid shell containing NBs may be formed. Bubbles occur when the size distribution is random. A well-suited covering material is filled in the bottle in a liquid state, and a specific gas is enclosed in the NBs. The bottle is mechanically agitated after fumes are introduced via the surface to encapsulate the required gas inside the material of the shell.^127^

###  Thin film hydration method

 One technique that is frequently used to produce lipid-encapsulated vesicles is thin-layer hydrating. First, formaldehyde was used to dissolve lecithin formulations. A rotary evaporator was then used to extract the organic solvent, creating a thin film. A lecithin-coated NB dissemination is obtained by sonicating or violently shaking the resultant film after it has been soaked in a buffer. By varying the shell’s makeup and the phospholipid membrane’s thickness in this system, it is possible to produce nanoscale bubbles.^128^

###  Cavitation method

 Cavitation is a well-known process for creating gas-laden bubbles. When a uniform liquid phase suffers a phase shift due to a quick pressure decrease below some critical value, void formation typically occurs. There are two methods of reducing pressure associated with fluid flow and sound fields, called hydrodynamic cavitation and acoustic cavitation, respectively.^129^

###  Hydrodynamic cavitation

 Evaporation or foaming occurs when a moving fluid is decompressed. As the local pressure increases, the generated gas bubbles collapse, creating a hydrodynamic void. By adjusting the temperature and pressure supplied to the flowing liquid, it is possible to regulate the size of the bubbles that are produced. Different system geometries, like ventures, orifices, and throttle valves resulting in velocity changes within the system, can produce hydrodynamic cavitation. A venturi system consists of three main parts: the inlet, the tube, and the conical outlet.^130,131^ During the operation of the system, both gas and liquid are concurrently transported through the venturi, and the narrow diameter of the conical convergence zone accelerates the flow velocity, thus reducing the pressure that causes cavitation. Using a venturi tube, we were able to generate NBs with average diameters from 130 to 529 nm from the air in water.^132^

###  Acoustic cavitation

 Acoustic cavitation can be caused by the propagation of ultrasonic waves in liquids. This causes pressure fluctuations and subsequent bubble formation. In order to separate dissolved gases, US uses localized compression-expansion cycles to create gas bubbles at pressures that are much below the saturated vapor pressure.^133^ NBs were produced from CO_2_ gas with a regular size of 300–500 nm by sonication in an aqueous system. The air NBs produced have a 750 nm diameter and may remain stable for an hour. Injecting air under pressure onto an aqueous medium to form a gas-supersaturated liquid and then using ultrasonic to it can also produce numerous gas bubbles.^134^

## Typical application of NBs

 Minimal gas-filled bubbles known as NBs, also known as nanoscale bubbles or ultrasonic contrast compounds, cover a size from a few nanometers to hundreds of nanometres. Their therapeutic applications are still being researched and explored, but they show promising potential in several areas. While NBs are promising for various therapeutic applications, it is important to note that further research and development are ongoing to optimize their efficacy, safety, and clinical utility.^135^

###  Photoacoustic/ultrasound imaging

 In US imaging, NBs can be employed as contrast agents to improve the visibility of tissues and organs. Due to their small size, NBs can circulate through the bloodstream and reach small blood vessels, enabling improved imaging of tumors, blood flow, and other diagnostic purposes. US imaging creates images based on how vibrations communicate with the environment using sound waves over 20 kHz. Skin and bone are examples of tissues and tissue separations that reflect, scatter, or receive ultrasonic waves (1-20 MHz) sent out through a transducer. The duration and power of the reflections can be used to establish the location of the tissue and its dispersion characteristics.^136^ Microbubbles, which consist of a rigid shell and vapor gist, are used as US contrast agents due to their high hindrance discrepancy and significant break up into the neighboring tissue. Bubbles reveal to an acoustic field experience spatial shaking and scatter.^137^

**Figure 9:**
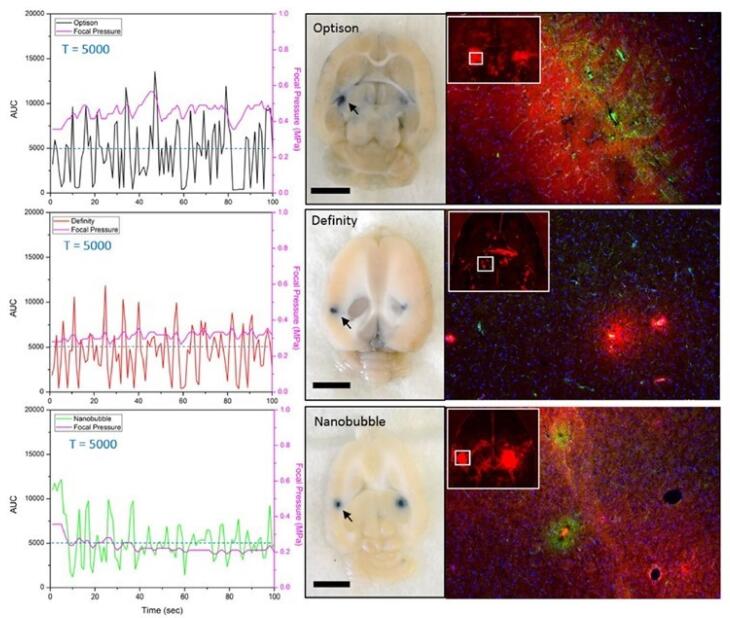
Demonstration of successful BBB opening with in-vivo feedback control based on the AUC responses for the 3 different agents. This figure is adapted from Bing et al^140^ under a Creative Commons Attribution 4.0 International License

 However, due to their size, microbubbles remain in blood vessels, limiting their clinical application. However, NBs are capable of getting through challenging biological barriers such as the border between the blood and the brain (BBB), malignant vessels, and cell membranes since they are smaller than the conventional microbubbles employed in clinical settings (Figure 9).^138–140^ As a contrast agent, NBs enhance US imaging, yielding finer details and more precise information crucial for accurately diagnosing malignant tumors. Metastatic and primary tumor imaging, as well as cerebral tumor border visualization, can be done simultaneously using acoustic and optoacoustic imaging.^141^ Real-life cancer diagnosis using photoacoustic technology is growing in popularity. In cancer ablation therapy, concurrent ultrasonography and visual acoustic imaging can be employed for the intraoperative examination of surgical borders and tumor borders.^142^ Key components of such medicines are extremely responsive to malignant cells and exact alignment between imaging modalities.^143^

###  Gene therapy

 NBs are useful for delivering genetic material such as DNA and ribonucleic acid (RNA) for gene therapy applications. By encapsulating genetic material in NBs, specific cells or tissues can be targeted, after which ultrasonic energy can be used to release the genetic material. This strategy has demonstrated potential in the therapy of cancer, genetic abnormalities, and numerous other diseases.

 In the field of drug delivery, the development of nucleic acid delivery systems is of great importance task. NBs underwent sonoporation to effectively improve the direct delivery of plasmid DNA into cysts. Sonoporation triggered cavitation effects that aided in gene transfer. The ability of NBs to transport genetic material was assessed in C26 cells by combining plasmid DNA with NBs to gauge their transfection capacity.^144^ Ultrasonic bombardment increased gene expression, indicating that NBs can help transfer genes. For the least-invasive in vivo propagation of genes, bladder lipid-based nanoparticles and ultrasonography are utilized together. Plasmonic NBs used in gene therapy directly interact with cells to transfect target genes. When plasmonic NBs form around gold nanoparticles, they absorb short laser pulses and generate heat, causing the nanoparticles to vaporize explosively. This accomplishes the dual task of cell death and external charge injection. The characteristics of the laser treatment and the gold nanoparticles control these characteristics. It has the advantage of delivering medicinal quantities of cells in a matter of minutes at an elevated evaluating rate of roughly 100 million cells per minute.^103,145-147^

 Gene therapy, which is gleaned from the capacity to deliver novel genetic material into the host, is a promising therapeutic strategy for treating inherited or acquired disorders. The inability to efficiently integrate bare nucleic acids is a key barrier to transferring genes. Due to their hydrophilic makeup, size, and having negatively charged phosphate groups, cells. Additionally, blood-based nucleases have the ability to degrade it. Numerous viral or non-viral gene delivery vehicles have been created in order to get around these restrictions.

 Nanoscale technologies were initially designed as contrasting substances, but they have only lately been investigated from the perspective of drug and gene delivery. Submicron-sized microbubbles, referred to as NBs,^148^ enable the transfection of CD3-positive cells using a combination of RNA interference (RNAi) and small carrier systems, offering cell type specificity with reduced cytotoxicity and enhanced efficiency in delivering therapeutic genes.

 Plasmonic NBs used in cellular modeling experiments show a single treatment strategy. Gene implantation with increased empathy, performance, and privacy. Optical destruction, heating, the creation of shock waves, and microbubbles are used in the gene transfer process.^103,147^ The establishment of non-viral gene delivery is crucial for gene therapy, and NBs have shown promise in this area. The two vectors were transfected into animal muscle tissues for experiments. With the help of US NBs, and positron emission tomography, it was possible to reliably see how genes were being expressed.^149^

###  Cancer treatment

 NBs have potential applications in cancer treatment approaches such as sonodynamic therapy (SDT). In SDT, NBs are combined with a photosensitizer and activated using US. This process generates reactive oxygen species that can selectively destroy cancer cells while sparing healthy tissue.

 In the therapy of cancer, NBs only serve as a carrier or in conjunction with US energy. The NBs produce a brief explosive reaction when exposed to laser energy, which adheres to tumor cells and releases the anti-cancer medication into the region that has been impacted. Both drug-sensitive carcinoma of the ovary and resistance to multiple drugs for breast cancer may benefit from it. Explosive NBs are made up of groups of gold nanoparticles that heat the surrounding water molecules, causing them to evaporate and create tiny bubbles that soon grow and burst. By causing epigenetic process modifications in hypoxic tumor cells, such as global 5mC-DNA methylation, oxygen NBs have been proven to be effective at treating malignancies.^150^ When injected intravenously, NBs are small enough to quickly pass through blood arteries and assemble at the tumor location. The enhanced permeability and retention effect, which is a hallmark of leaky tumor blood arteries with inadequate lymphatic drainage, causes this passive buildup.^151^

 Reactive oxygen species engendered by the NBs cause damage to the cancer cell membranes, proteins, and DNA. This oxidative stress leads to cell death and the destruction of the tumor tissue. Importantly, this therapy selectively targets cancer cells while minimizing damage to healthy cells and tissues.^152^

###  Hypoxia treatment

 NBs have potential applications in treating hypoxia, which is characterized by low oxygen levels in tissues. Research in this area has not yet been completed. NBs can serve as carriers for delivering oxygen to hypoxic tissues. By encapsulating oxygen gas within NBs, they can be injected into the bloodstream, where they circulate and reach areas with low oxygen levels. Once in the target tissue, the NBs can release oxygen, thereby supplementing the oxygen supply and alleviating hypoxia.^153^

 Two main strategies for oxygen delivery using NBs are (*a*) intravenous infusion of NBs followed by high-intensity US to disrupt the NBs, and (*b*) introduction of oxygen into a concentration gradient. to spread along. Availability of additive-free oxygen NB water for hypoxia of cancer cells. Hypoxia-inducible factor-1a concentrations in breast tumors and carcinomas of the lungs were measured to evaluate this mechanism. According to reports, oxygen NB water holds hope for combating the hypoxia-induced radiation tolerance of cancer cells.^125^

###  Atherosclerosis treatment

 Atherosclerotic plaque tissue can be removed with short laser pulses without damaging (thermally or mechanically) normal tissue. Plasmonic at the microscopic level in this therapy, NBs exhibit selective, precise, and superior tissue ablation. It has the potential to be used as a new generation of curative instruments. Laser pulses can trigger NBs without causing significant photothermal consequences. Coronary artery plaque is delivered in 1 to 10 laser pulses that have an unequivocal laser fluence. This perspective is scrupulous, non-thermal, and aids in removing atherosclerotic plaque. NBs serve as contrast agents for imaging methods such as US and other imaging methods. It improves the visualization of atherosclerotic plaques and provides valuable information on their location, size, and composition. This helps early detection and diagnosis of atherosclerosis, allowing for timely intervention and treatment.^154,155^

## Route of administration

 The NB delivery route refers to the delivery of therapeutic drugs or agents using NBs as carriers. Compact gas-filled bubbles having diameters in the nanometer range are called NBs. They are appealing for several biomedical applications, including medication administration, because of their special qualities. NBs can be designed to encapsulate drugs, nucleic acids, or other therapeutic substances. These are typically designed to be stable and transport payloads to specific destinations within the body. NBs can be administered in a variety of ways, depending on the specific application and desired therapeutic outcome. Numerous types of shells and cores have been used in different routes of administration of drugs.

###  Intravitreal administration

**Figure 10:**
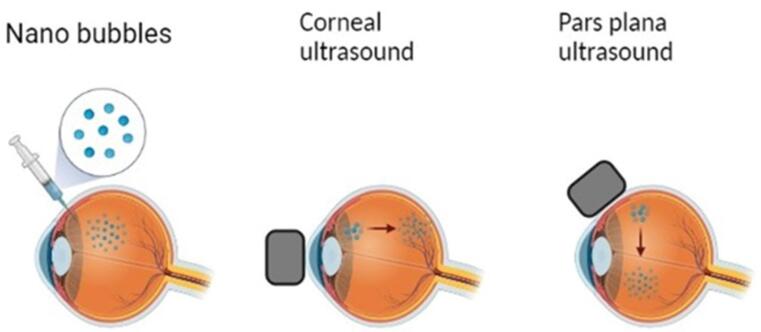
Intravitreal administration of NBs

 Due to the injected therapeutic agent’s non-specific distribution and early clearance from the ocular tissues, the intravitreal route faces many obstacles that prevent it from treating lesions in the posterior portion of the eye quickly and effectively (Figure 10). By altering the pattern of therapeutic particle movement, NBs, and US can enhance the effects of drugs in the vitreous.

 Nearly every section of the retina is affected by a variety of disorders, and intravitreal medication administration produces a higher therapeutic dose over these layers when compared to conventional dosing. The safe use of US-enhanced medication delivery in ocular tissues has shown that it can improve therapeutic penetration through the cornea and sclera. Additionally, preliminary trials on this approach are being done to see if it might improve intravitreal medication translocation.^156,157^

 Nearly every section of the retina is affected by a variety of disorders, and intravitreal medication administration produces a higher therapeutic dose over these layers when compared to conventional dosing. The safe use of US-enhanced medication delivery in ocular tissues has shown that it can improve therapeutic penetration through the cornea and sclera. Additionally, preliminary trials on this approach are being done to see if it might improve intravitreal medication translocation.^39^

###  Oral administration

 Orally administered NBs refer to the oral delivery of NBs, typically in the form of oral solutions or capsules. This route of administration has the advantage of its non-invasiveness and ease of use. However, the harsh conditions within the gastrointestinal tract, such as the acidic environment of the abdomen and the presence of digestive enzymes, pose challenges to this. Once orally administered as liquids or encapsulated in solid dosage forms like capsules or tablets, NBs encounter acidic pH and mechanical agitation within the stomach. The stability of NBs under these conditions is pivotal for their effective oral delivery. This delivery method holds promise for various applications, including targeted drug delivery to the gastrointestinal tract, managing gastrointestinal disorders, and facilitating systemic drug delivery.^158^

 A suspension of oxygen NBs maintained by a surfactant was supplied orally in experiments to reduce tumor hypoxia. Tests were conducted on mouse tumor models for human pancreatic cancer. 100 μL oxygen-saturated water, oxygen NBs/argon NBs were forcefully given to animals, and expression of a hypoxia-induced factor was measured which shows a significant change in their transitional level.^36^

###  Intravenous administration

 NBs can be injected directly into the bloodstream by intravenous injection. This pathway allows for systemic distribution of NBs and their payloads throughout the body. They may reach target tissues or organs, such as tumors, where they may release therapeutic agents. Microbubbles floating in liquids can function as incredibly effective US reflectors when there is a central gas core present. Internal organ visualization is made easier by infusing a water-based suspension of air microbubbles into the bloodstream, which considerably improves contrast obtained by ultrasonic echo-graphic imaging. It has been shown to help detect cardiovascular and other diseases. The common diameter range of microbubbles employed in clinical imaging is between 1 and 10 m (the maximum limit for transit via the lung capillaries), however, these sizes also restrict extravasation of the microbubbles from the bloodstream. Typically, a microbubble’s diameter is comparable to that of a red blood cell, leading to similar viscosity in the body’s capillaries and microvessels after intravenous delivery.^159^

## Theranostic view

 NBs have emerged as a promising therapeutic platform in recent years. Theranostics integrates diagnostic and therapeutic functions into one system, enabling simultaneous monitoring and treatment of disease.

###  Theranostic in cancer treatment

 Cancer theranostic is a new medical term that combines the words treatment and diagnosis.^160^ The close relationship between diagnosis and treatment in theranostics has opened the door to accurate identification and customized treatment for malignant patients. Most importantly, Theranostics has the following benefits: (*a*) after therapy, to be able to see and track the effectiveness of anti-cancer medications on the patient’s tumor. (*b*) capability to treat patients immediately, specifically, and individually (*c*) ability to concurrently recognize and treat patients with early-stage cancer. As an outcome, it is possible to reduce cancer-related fatalities, particularly in cancer patients who are near the end of their life.

 In order to precisely identify and treat malignancy, modern imaging methods with highly sensitive probes must be developed. Therapeutic substances such as plasmonic NBs, radioisotopes, liposomes, and quantum dots. can be coupled with neoplastic medications, malignant cell markers, and imaging technologies, which have the potential to enhance the identification, therapy, and supervision of cancer patients.^161^

**Figure 11:**
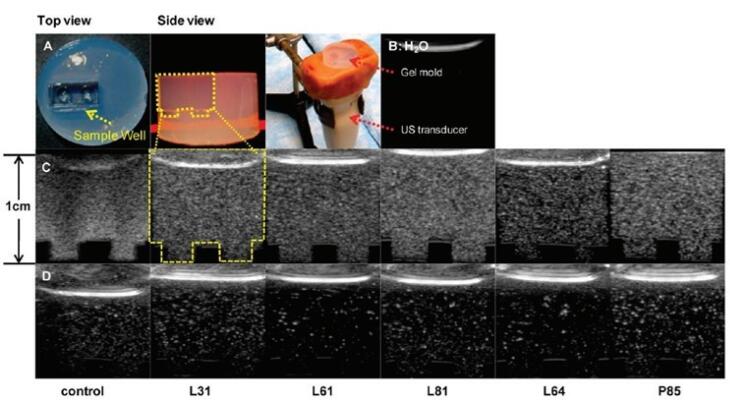
Representative control, Pluronic bubble grayscale ultrasound images in vitro in custom-made agarose gel mold and experimental setup P85 at equivalent bubble concentrations. Reprinted with permission from Krupka et al^163^; Copyright (2014) American Chemical Society.

 Recently, plasmonic/Pluronic NBs have been identified as possible therapeutic agents for the treatment of cancer (Table 4, Figure 11). Nanoparticles coupled to cell-targeting particles, which include antibodies or ligands, are introduced into the human body, where they cling to and infiltrate the target cancer cells.^162,163^

**Table 4:** Application of plasmonic NBs

**Plasmonic nanobubble-based agent**	**Application**	**Reference**
Plasmonic gold nanostar probe for photothermal therapy	Treatment inhibited high-grade sarcoma growth; CT tracked plasmonic nanobubble accumulation.	^164^
Magnetic core-star-shaped gold plasmonic shells conjugated with SK-BR-3 breast cancer-targeting S6 aptamers	S6 aptamer on plasmonic envelopes increased accumulation on SK-BR-3 breast cancer cells. Gold nanoparticles induced cell death under near-infrared radiation.	^165^
Plasmonic NB with gold nanorod	Plasmonic NB-derived gold nanorods harmed lung cells under laser.	^166^
Multifunctional magneto-plasmonic CoFe204@Au core-shell nanoparticle with doxorubicin	HEp2 hepatocellular carcinoma cells' viability decreased under 2.5 GHz microwaves.	^167^
Plasmonic nanobubble coupled with poly(sodium-4-styrene sulfonate) (PSS), doxorubicin (DOXO), poly-1-lysine hydrobromide (PLL)hyaluronic acid (HA)-coated	Near-infrared radiation triggered cytotoxicity and apoptosis in HeLa cervical and MDA-MB-231 breast cancer cells.	^168^

###  Magnetic nanobubble

**Figure 12:**
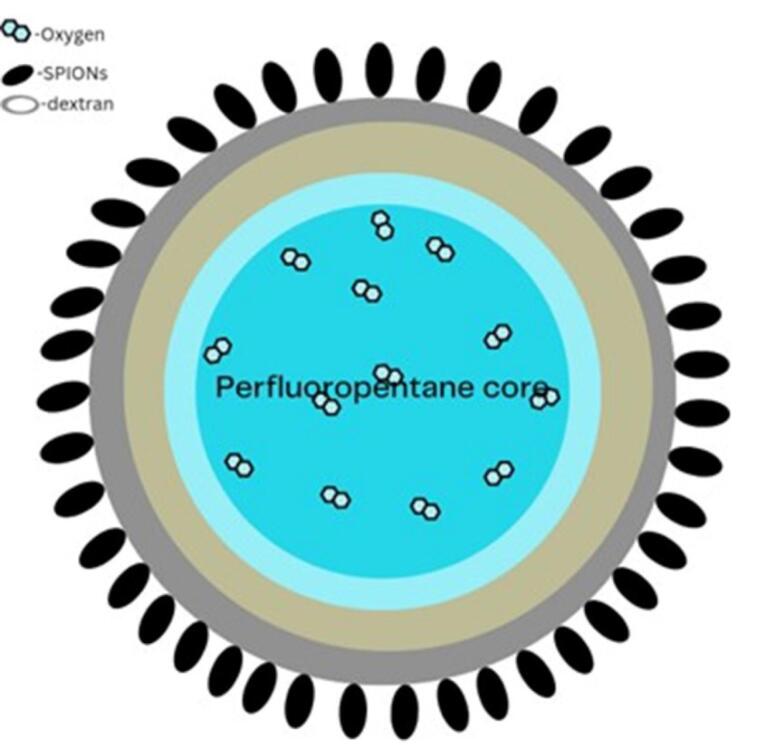
Structure of Magnetic NB

 Researchers have looked into using magnetic oxygen-loaded NBs (MOLNBs), which are made by adding superparamagnetic iron oxide nanoparticles to the surface of polymeric NBs, as therapeutic carriers for the treatment of cancer and oxygenation of brain tumors (Figure 12). *In vitro*, internalization by human brain microvascular endothelial cells, photophysical and cyto-toxicological attributes, and mobility of MOLNB in a fixed magnetic field were all investigated. MOLNB is a safe oxygenation vector that crosses cerebral membranes and is propelled through the central nervous system to deliver shipments to places of particular heed. Additionally, MOLNB can be monitored using MRI US or ultrasonography. MOLNB has potential applications in the treatment of brain malignancies since it can improve traditional radiation therapy and give cancer treatment that is guided by ad hoc magnetic attraction modified under MRI and/or US monitoring.^169^

###  Malaria theranostic nanobubble

 The presence of drug resistance and the severe impact of malaria infections pose significant challenges in diagnosing and treating this persistent and life-threatening human disease. Overcoming this issue demands early detection strategies and innovative approaches for effective parasite eradication. We used hemozoin crystals’ strong light absorption and nanosized to mechanically identify and eliminate malaria parasites with one treatment procedure. Light scattering signals from NBs indicate the presence malaria parasite. The same NB mechanical action physically destroys parasites in nanoseconds without the use of drugs. *In vitro*, laser-persuaded NB therapies can eliminate up to 95% of parasites, and they are 8 times more effective at doing so than standard chloroquine drug therapy. The destruction method is very selective for erythrocytes infected with malaria and does not harm nearby erythrocytes that are not infected. Because of this, the creation of vapour NBs surrounding hemozoin by laser pulses facilitates the quick and extremely accurate identification and elimination of malaria parasites during treatment procedures.^170^

###  Industrial challenges associated with NB formulation

 Industrial challenges associated with NBs in pharmaceutical applications encompass controlled generation, stability, scale-up, characterization, contamination control, and regulatory compliance. Generating NBs consistently and in controlled sizes is arduous due to their sub-micron dimensions, necessitating precise equipment and process parameters to regulate nucleation and growth.^171^ Moreover, NBs’ inherent instability, prone to coalescence or dissolution, mandates meticulous maintenance to uphold stability over time for pharmaceutical formulations. Scaling up production from lab to industrial scale poses significant hurdles, as processes must ensure consistent quality and yield while remaining cost-effective. Characterizing NBs accurately is challenging due to their small size and transient nature, requiring specialized analytical methods for quality control and optimization. Maintaining a sterile environment to prevent contamination during NB introduction into formulations necessitates stringent control measures and specialized equipment. Finally, regulatory compliance demands comprehensive testing and documentation to demonstrate the safety, efficacy, and stability of NB-based pharmaceutical products for obtaining regulatory approval. These challenges underscore the complexities inherent in leveraging NBs for pharmaceutical applications and emphasize the need for innovative solutions and interdisciplinary collaboration to address them effectively.

## Clinical trials

 NBs find application in pharmaceuticals, particularly in clinical studies demonstrating their responsiveness to US for delivery purposes.^172,173^ The controllability of drug and genetic material delivery and therapeutic visualization using ultrasonic switches was supported by the results of validated clinical studies. Numerous preclinical studies have also displayed that the use of microbubbles in conjunction with US may improve medication delivery, gene expression, or both in certain targeted areas. FDA-approved drugs and drugs under clinical trials have been reported in (Table 5).

**Table 5:** List of Nanobubble medications under clinical trials and in market

**Drug**	**Status**	**Application**	**Reference**
Albunex	Marketed	Photoacoustic imaging	^174^
Perflutren lipid (Definity)	Marketed	ECG	^175^
Sonazoid	Marketed	US imaging	^176^
Sonovue	Marketed	US imaging	^177^
Lumason	Marketed	US imaging	^177^
MRX 815	Phase II trials	Sonothrombolysis	^178^
RNS60	Clinical trials	For treating Traumatic brain injury & amyotrophic lateral sclerosis	^179^
Doxorubicin	Marketed	Chemotherapy	^180^
Methotrexate	Marketed	Chemotherapy	^181^
Sorafenib	Marketed	Chemotherapy	^182^
Porphyrins chemical pigment	Marketed	Chemotherapy	^183^
Doxorubicin and Ciclosporin A	Marketed	Chemotherapy	^184^
Pemetrexed and Pazopanib	Marketed	Chemotherapy	^185^
Resveratrol	Marketed	Anticatabolic & cell apoptosis	^186^
Biotinylated anti-ErbB2 Affibody	Marketed	Inhibits overexpression of HER2 gene, ovarian cancer, lung cancer	^187^
Paclitaxel	Marketed	Treating prostate cancer	^188^
Vancomycin	Marketed	Antibiotic	^189^
Apomorphine	Marketed	Anti-Parkinson’s	^190^

 Preclinical studies in a xenograft mouse model showed that integrated treatment with doxorubicin-loaded glycol chitosan NBs enhanced the antitumor effects of doxorubicin and prevented drug-induced cardiac injury in acute traumatic coagulopathy.^191^ Apatinib, a new selective VEGF receptor 2 inhibitor, has shown promise as initial therapy for patients with advanced hepatocellular carcinoma, according to a number of preclinical and clinical studies. Participants with advanced solid tumors participated in Phase 1 research with a maximal acceptable quantity of 850 mg once a day. Apatinib significantly increased PFS compared to placebo in a phase II study involving people with metastatic gastric cancer. Hand-foot syndrome, tiredness, and hypertension were among the side effects, however, they were successfully treated.^192^ A recent clinical study validated the beneficial effects of NB water applied to curcumin extract to enhance explosive power without exercise therapy. Nanobubble water curcumin extract supplements have anti-fatigue effects and are useful when performing explosive or intense exercise, including general physiological protection. In particular, it reduces the risk of injury in drop jumping for girls.^193^

## Quality by design

 QbD is a coherent approach to drug development that focuses on ensuring product quality through design and controlling critical variables throughout the development process. QbD principles are typically applied to the development of pharmaceutical formulations, but can also be extended to the development of NBs, especially in the context of drug delivery systems and nanotechnology applications. Risk assessment helps identify hazards, drug interactions, toxicity, and properties that can affect product quality.^194^

###  Quality target product profile

 QTPP describes NB products’ desired properties and performance, including size distribution, stability, surface charge, drug loading capacity, and targeted delivery properties (Figure 12). This step helps define CQAs that need to be controlled during development. A protective barrier with hydration qualities is created by surfactant molecules adhering to the gas-water interface of NBs, improving their stability.^195^

###  Critical quality attributes

 CQA is a parameter that directly affects the safety, efficacy, and quality of NBs. These include attributes such as size, stability, surface charge, drug release kinetics, and biocompatibility. He must identify and prioritize CQAs based on their impact on end-product performance.

 Although many advances have been made in improving the stability of NBs, there is still a need to further optimize NB formulations to promote the in vivo circulation half-life. Increasing the in-plane stiffness of the lipid monolayer has been shown to slow gas diffusion into the surrounding medium and reduce bubble dissolution. Enhanced in-plane stiffness can be achieved by increasing the length of lipid acyl chains in the bladder membrane. Longer acyl chains have been shown to increase van der Waals attraction between lipids and improve bladder stability by increasing the flexural modulus.^196,197^

 There is no reliable methodology to produce lipid-based NBs with the same level of consistency in size as protein-based NBs. Extrusion is frequently utilized for size reduction and size standardization of nanoparticles. The extruded NBs had a 3-fold increase in the average polydispersity index of the NBs and also increased echogenic properties compared to the raw NBs.^39,198^

 Recently, Ahmed et al proposed absolute ZP values of -30, -36, and -45 mV for three NBs via air, O2, and N2 gases using SDS as an anion-based surfactant at 500 mg/L I understand increased to -65, 60, or -80 mV.^199^

###  Critical material attributes

 Electrocatalytic NB generation was achieved after considerable supersaturation of the gas on the electrode surface. This development method improves stability and increases the nucleation process.^200-209^

 The CMA show which aspects of the material have an impact on the important quality qualities. They must therefore be regulated or monitored to guarantee the quality of the desired medicinal product. NBs with a diameter of smaller than 1 μm have special physicochemical characteristics, including a low maximum speed, a large specific surface area, and a long life.^35,130,210^

 Nazari et al presented that NB produced in the existence of dodecyl amine (DDA) proved to be more enduring than pine oil (PO), methyl isobutyl carbinol (MIBC), and dipropylene glycol (DPG).^211^ Yasui et al and Zhou et al confined that certain dodecyl amine may absorb NBs’ surface which makes them more positive charge.^212^ Numerous studies have revealed the significant role that NBs spawned by MIBC, PEB70, and F10-YS-20 play in flotation.^213,214^ The adsorption actions of bulk NB prepared by cavitation caused by hydrodynamics on mica surfaces in the presence of DDA were reported by Zhou et al which states that in the existence of DDA, ultrafine vesicles adsorbed to the muscovite surface, making the surface furthermore hydrophobic and ultimately increasing flotation efficiency by about 18%.^82^ Alkyl trimethyl ammonium bromide is added to pure water, and then the application of US was shown to reduce the NBs size from 750 nm to 450 nm.^215^

###  Critical process parameter

 Critical process parameters (CPP) refer to certain variables or conditions that have a significant impact on the manufacturing process and directly affect the quality characteristics of the product. These parameters must be tightly controlled within predefined limits to ensure a consistently high-quality product. CPPs are typically identified during process development and are critical to ensuring process robustness, efficiency, and reproducibility. Examples of CPPs in pharmaceutical manufacturing: (*a*) temperature and humidity during pharmaceutical synthesis or formulation, (*b*) Mixing speed and time during the mixing process, and (*c*) Pressure and flow in filtration and sterilization processes.

 Nucleation sites provide a surface for bubble formation. These sites can be created in various ways, such as incorporating microbubbles or using materials with specific surface properties. NB formation is significantly influenced by nucleation sites. NBs are known to occur more frequently on hydrophobic surfaces in water. The formation of NB requires gas supersaturation in aqueous solutions. Three acknowledged hypotheses of gas nucleation are as follows: The theory on Harvey cores, free gas cores, and vapor cavity cores.

###  Harvey nuclei

 The presentation and use of a sophisticated cell nucleation strategy to polymer frothing procedures. The analysis of bubble nucleation experiments makes use of the overall energy equilibrium and Clausius-Duhem inequality. The work, surface tension, and critical work of the creation of clusters are factors of the Hamaker stable, the interaction distance between molecules and/or repeat units, the mole fraction of each component, the cluster radius, and the mole fraction of every constituent, according to calculations of intermolecular long-range potentials.^216^

###  Free gas nuclei

 A given set of conceivable system states can be eliminated from thermodynamic equilibrium states by using free energy minimization. However, it occasionally happens that the selected free energy function reaches an inaccurate minimum that is inconsistent with the Young contact angle criterion, particularly for capillary systems.^217^

###  Vapor cavity nuclei theory

 Homogeneous nucleation theory prognosticates that the tensile strength of water is much greater than observed. Plesset suggested that this could be due to the presence of solid impurities and performed special calculations for hydrophobic spheres. We find that (*i*) the radius of the sphere exceeds 10 Å and (*ii*) the thickness of the vapor shell surrounding the sphere must be at least equal to the average intermolecular distance of about 3 Å, his results suggesting the possibility of cavitation. indicates that it is low. water. Since uniform nucleation is believed to be effective in producing vapor phases with radii up to about 10 Å, the presence of smooth spheres of arbitrary size, hydrophobic or not, allows the predicted tensile strength can be reduced by up to 30%.^218^

 Air bubbles separated by centrifugation were found to be highly echogenic. All were less than 1 μm in diameter, with the majority ranging from 100 to 600 nm. If filtering with polycarbonate membranes was found to correlate with loss of US signal. The pore size of the membrane becomes smaller. However, US signals were observed in all of them. Conditions that indicate the presence of bubbles in the ranges 100-400 nm, 400-600 nm, and 600-1000 nm, contribute to the visible contrast at this ultrasonic frequency. The difference in clarity between the filtered solutions was because the bubbles became smaller and there were fewer of them. This made it necessary to study them separately to understand their effects better.^219^

 Sonication plays a crucial role in forming vapor NBs. Ultrasonic vibrations of wetted surfaces can cause rapid pressure fluctuations within the liquid, and gas bubbles can form in localized regions below the vapor pressure.^220-223^ Recently, it was discovered that very high-frequency vibrations can also form nanoscale bubbles upon heating when the local temperature exceeds the boiling point of the liquid.^224^

## Futuristic aspects

###  New image analysis method for cancer detection with an US contrast agent

 NBs may offer advantages in post-imaging image processing that may improve disease detection and visualization. Current clinical protocols can use microbubbles to image blood vessels, but NBs can accumulate within the tumor parenchyma, providing new imaging opportunities. NBs can extravasate, making them useful for imaging tumor tissue rather than just blood vessels. Visualizing tumor parenchyma’s size and shape can help with surgical planning, illness diagnosis, and prognosis. To achieve this, advancements in imaging may involve modified versions of existing techniques, like utilizing MIP (maximum intensity projection) imaging. For US contrast imaging, a maximum intensity persistence algorithm should be applied at each image pixel to monitor the growth of air bubbles inside the tumor.^225^

###  Improved detection of prostate cancer with PSMA-targeted US contrast agents

 NBs have potential applications in molecular imaging for detecting cancers such as ovarian and prostate cancer, both commonly diagnosed using US imaging. In particular, transrectal US-guided biopsy is the standard of care for these patients. However, these procedures are essentially patterned and not case-specific, as US imaging can image only the prostate and cannot delineate cancer within the prostate. Contrast-enhanced ultrasound (CEUS) has been shown to improve prostate cancer detection. A recent report found that transrectal CEUS-guided biopsy was more sensitive than random biopsy for detecting prostate cancer in men with a previous negative biopsy and positive multiparametric MR imaging findings are suggested.^226^

## Conclusion

 NBs heralded for their potential in targeted drug delivery and imaging, represent a promising frontier in chemotherapy for malignant neoplasms. The evolution of chemotherapy demands continuous evaluation to enhance drug effectiveness while minimizing systemic toxicity. Innovative techniques like US-targeted NB destruction hold immense promise, enabling precise drug delivery to solid tumors while reducing systemic exposure. However, challenges persist, such as the low payload efficacy of lipid-encapsulated NBs and limitations in molecular targeting due to avidin’s immunogenicity. Additionally, cores in US-responsive materials occupying significant space hinder effective drug loading, impacting therapeutic potential. Despite the advantages offered by NBs in precise drug delivery and imaging, addressing payload efficacy, targeting constraints, and the delicate balance between therapeutic effectiveness and potential harm to healthy cells remains imperative for broader clinical application in cancer treatment and diagnostics. Advancements focusing on enhancing drug-carrying capacities and refining targeting methods will drive progress in this domain, ultimately benefiting cancer patients and advancing diagnostic imaging techniques.

## Acknowledgments

 The authors would like to express their administration to the College of Pharmacy, SRMIST for providing them with tremendous opportunities to accomplish this work.

## Competing Interests

 The authors declare no conflicts of interest.

## Data Availability Statement

 The datasets generated during and/or analyzed during the current study are available from the corresponding author upon reasonable request.

## Ethical Approval

 This article does not contain any studies with human participants or animals performed by any of the authors.
